# The diet of a nocturnal pelagic predator, the Bulwer’s petrel, across the lunar cycle

**DOI:** 10.1038/s41598-017-01312-3

**Published:** 2017-05-03

**Authors:** S. Waap, W. O. C. Symondson, J. P. Granadeiro, H. Alonso, C. Serra-Gonçalves, M. P. Dias, P. Catry

**Affiliations:** 1 Cardiff School of Biosciences, Sir Martin Evans Building, Cardiff University, Museum Avenue, Cardiff CF10 3AX UK; 2 MARE-Marine and Environmental Sciences Centre, ISPA-Instituto Universitário, Rua Jardim do Tabaco 34, 1149-041 Lisboa, Portugal; 3 Centre for Environmental and Marine Studies, Departamento de Biologia Animal, Faculdade de Ciências, Universidade de Lisboa, Campo Grande 1749-016 Lisboa, Portugal; 4 Institute of Marine Research (IMAR/CMA), Department of Life Sciences, University of Coimbra, 3004-517 Coimbra, Portugal; 5 Faculdade de Ciências da Universidade de Lisboa (FCUL), Campo Grande 1749-016 Lisboa, Portugal; 6 BirdLife International, The David Attenborough Building, Pembroke Street, Cambridge, CB2 3QZ UK

## Abstract

The lunar cycle is believed to strongly influence the vertical distribution of many oceanic taxa, with implications for the foraging behaviour of nocturnal marine predators. Most studies to date testing lunar effects on foraging have focused on predator activity at-sea, with some birds and marine mammals demonstrating contrasting behavioural patterns, depending on the lunar-phase. However, to date no study has focused on how the lunar cycle might actually affect predator-prey interactions in the upper layers of the ocean. Here, we tested whether the diet of the predominantly nocturnal pelagic predator, the Bulwer’s petrel (*Bulweria bulwerii*) would change throughout the lunar cycle, using molecular analysis to augment detection and taxonomic resolution of prey collected from stomach-contents. We found no evidence of dietary shifts in species composition or diversity, with Bulwer’s petrel always consuming a wide range of mesopelagic species. Other co-variables potentially affecting light availability at-sea, such as percentage of cloud cover, did not confound our results. Moreover, many of the species found are thought not to reach the sea-surface. Our findings reveal that nocturnal predators are probably more specialized than previously assumed, irrespective of ambient-light, but also reveal deficiencies in our current understanding of species vertical distribution and predation-dynamics at-sea.

## Introduction

It is well known that the moon can affect animal behavior and reproduction^1^. During full moons nocturnal animals might either increase activity, taking advantage of visual cues to mate and find food, or reduce activity to avoid predators^2, 3^. The moon further exerts an important influence on environmental factors by creating tides, so that many marine species have developed lunar periodic rhythms of 14.8 days and 29.5 days to optimize foraging, reproduction and dispersal^4–6^.

In the deep scattering layers of the ocean, many animals respond to solar light intensities, migrating upwards in the water column at night to feed closer to the surface, while descending to deeper layers during the day to avoid predators^7^. However, species of the deep scattering layers also react to changes in moonlight intensity, migrating closer to the surface during new moons than during full moons^8–11^. Such cyclical responses of mesopelagic organisms to moonlight are thought to induce significantly different foraging strategies in predators.

Imber^12^ suggested that many pelagic predators have lower foraging success during full moons, as many of their prey are known to remain at greater depths in what represents an escape response to light. Such a hypothesis has been suggested to explain, for example, lower at sea activity in swallow-tailed gulls (*Creagrus furcatus*) and deeper foraging dives by fur seals (*Arctocephalus galapagoensis*) off the Galapagos Islands during moonlit nights^13, 14^. Despite the fact that substantially fewer prey are thought to be available at the sea surface during full moon, other taxa remain close to the surface during the day (e.g. epipelagic species) and probably do not react to moonlight intensity across the lunar cycle. Hence, predator-prey relationships and energy and mass transfers in the upper layers of the ocean might change along the lunar cycle.

Despite known effects of the moon on the vertical migration patterns of species of the deep scattering layers, and its potential effects on ecosystem bottom-up and top-down processes^15, 16^, this topic has received very little attention in ecological studies, except for speculative theories concerning changes in foraging efficiency across the lunar cycle based on the activity patterns of predators obtained by data loggers^14, 17, 18^. To date almost nothing is known about the range of deep scattering layers species that react to moonlight, how moon-induced activity patterns relate to prey consumed, and what the implications may be for the foraging choices and success of predators.

Dietary studies on marine top predators may reveal the impact of environmental factors on prey species availability, while providing insight into demographic regulation of predator populations, the structure of food webs and the organization of communities. Birds play a major role in marine trophic webs, primarily due to their important function in ecosystem regulation, with, for example, ca 70 million tones of the ocean’s biomass being consumed annually by seabirds^19^, approaching the global catch by marine fisheries^20^.

Various techniques have been developed to analyse the diet of seabirds, including morphological analyses of stomach-contents^21, 22^, stable isotopes^23^ and more recently molecular techniques applied to faeces and stomach-contents. Despite being extensively applied in dietary analysis of terrestrial vertebrates^24–26^, molecular methods have rarely been used to assess the diet of seabirds and have only been applied to a few taxa including penguins (*Eudyptes chrysolophus*; *Pygoscelis adeliae*)^27–29^, puffins (*Fratercula arctica*)^30^ and Cory’s shearwater (*Calonectris borealis*)^31^. Prey species composition obtained through molecular methods provides higher taxonomical resolution, where target genes possess variable regions that are distinguishable at the species level (e.g. the cytochrome oxidase 1 [COI] barcoding region). Prey identification based on commonly used and internationally agreed barcodes, such as COI, identify more species due to higher representation of sequences in public databases (GenBank and BOLD^32^), although in some marine taxa better representation can be found using the 16S gene (see below). This facilitates assigning names to prey species without any *a priori* knowledge of the prey range consumed by predators. This contrasts with morphological techniques, based upon incomplete regional reference collections that often achieve lower levels of discrimination and miss taxa without hard parts.

Here, we assessed the influence of the lunar cycle on the diet of a small pelagic seabird, the Bulwer’s petrel, *Bulweria bulwerii*, using molecular diagnostics to assess changes in prey composition in relation to the lunar cycle.

Bulwer’s petrels are shallow divers, reaching at most around 5 m depth^33^. Previous studies of Bulwer’s petrels showed predominantly nocturnal flight activity at sea^34, 35^ and a high reliance on mesopelagic prey^36–38^, although some studies also report consumption of surface prey^39^. Given that mesopelagic prey are generally found in deeper oceanic layers, usually at depths below 200 meters, such prey are hypothesized to become available to Bulwer’s petrels only at night, when species of the deep scattering layers ascend towards the water surface to feed. Moonlight might therefore exert an important negative effect on the abundance and range of prey species available to Bulwer’s petrel.

Based on current knowledge of the vertical movements of mesopelagic fish and squid, and of the diet and behavioural patterns of their avian predators, we would hypothesize that, during periods of greater moonlight illumination:

1. Bulwer’s petrels would partly shift their diet towards epipelagic prey, to compensate for a scarcity of their usual mesopelagic prey.
2. The mesopelagic dietary components would show a decline in diversity, being mostly based on fewer species less responsive to nocturnal illumination.

## Results

### Prey identification of chicks using molecular analysis

In total, 988 prey items (vertebra and tissue) were collected from 139 stomach-contents of Bulwer’s petrels. The combined use of morphological analysis on hard part remains and molecular analysis of 16S rRNA and COI barcodes revealed that these prey items corresponded to a minimum of 384 different individual prey.

Morphological analysis of vertebra only revealed 15 distinct taxa, of which seven were identified to genus and species levels, two to families, while six remained unidentified. Molecular analysis substantially augmented prey detection and resolution, revealing 73 distinct taxa: 50 different fish identified using BOLD and 23 distinct cephalopods showing separate clusters on the tree (Fig. S1). Of these, 61 were positively assigned to genera and species and 8 to family ranks. Positive taxonomic assignments using each method, molecular- and morphological-based analyses, are indicated in Table 1. Although molecular analysis substantially outperformed morphological taxonomical identifications, it is noteworthy that DNA extracted from the tissue of *Diretmus argenteus* and *Argyropelecus* often failed to produce positive amplifications during PCR, however, these were effectively identified using morphological analysis of vertebra. Both of these prey items were usually of very small sizes (1–2 cm) and had small quantities of tissue attached, which might have compromised the quality of the DNA extracted.

**Table 1:** Frequency of occurrences (%FO) of prey in the stomach-contents of chicks of Bulwer’s petrel.

Order	Family	Taxa	Similarity (%)	Total	Full Moon	New Moon	Quarter
**Cephalopoda**							
Oegopsina	Architeuthidae	Architeuthis dux* ^**g**^	91.90–94.50	11.51	19.23	10.34	0
	Bathyteuthidae	*Bathyteuthis abyssicola* ^**g**^	96.93	1.44	1.92	1.72	0
	Chiroteuthidae	Undientified^**g**^	94.51	0.72	0	1.72	0
		*Grimalditeuthis bonplandi* ^**g**^	100	0.72	0	1.72	0
	Cranchiidae	*Helicocranchia pfefferi* ^**g**^	96.03	0.72	0	1.72	0
		*Leachia* ^**g**^	96.15–96.22	11.51	11.54	13.79	7.14
		*Taonius pavo* ^**g**^	98.60–99.2	2.16	1.92	3.45	0
		Unidentified^**g**^	94.28	0.72	0	1.72	0
	Cycloteuthidae	*Cycloteuthis sirventi* ^**g**^	96	1.44	0	1.72	3.57
	Histioteuthidae	*Histioteuthis* OTU1^**g**^	96.61–97.13	7.91	7.69	6.9	10.71
		*Histioteuthis* OTU2^**g**^	97.56	2.16	1.92	3.45	0
		*Histioteuthis* OTU3^**g**^	99.7	0.72	1.92	0	0
		*Stigmatoteuthis cf hoylei* ^**g**^	99.18–99.59	31.65	40.38	24.14	32.14
		*Histioteuthis reversa* ^**g**^	99.59	3.6	1.92	6.9	0
	Joubiniteuthidae	*Joubiniteuthis portieri* ^**g**^	99.55	1.44	1.92	1.72	0
	Lepidoteuthidae	*Lepidoteuthis grimaldii* ^**g**^	98.97	1.44	0	3.45	0
	Mastigoteuthidae	*Mastigoteuthis magna* ^**g**^	99.6	0.72	0	1.72	0
		*Mastigoteuthis hjortii* ^**g**^	97.69	5.04	5.77	5.17	3.57
	Octopoteuthidae	*Octopoteuthis megaptera* ^**g**^	95.39	0.72	1.92	0	0
		*Taningia danae* ^**g**^	93.07	0.72	1.92	0	0
	Ommastrephidae	*Ommastrephes bartramii* ^**g**^	99.8	5.04	1.92	3.45	1.43
	Onychoteuthidae	*Onykia sp* ^**g**^	98.31	0.72	1.92	0	0
	Unknown Teuthida	unknown Teuthida^**g**^	92.28	0.72	1.92	0	0
**Teleosts**							
Anguilliformes (eels)	Synaphobranchidae	Unidentified^**m**^		0.72	0	0	3.57
	Derichthyidae	*Derichthys serpentinus* ^**g**^	100	0.72	1.92	0	0
Argentiniformes (marine smelt and related)	Microstomatidae	Unidentified^**g,m**^	98.77	2.16	1.92	1.72	3.57
	Platytroctidae	*Searsia koefoedi* ^**g**^	99.66–99.82	0.72	1.92	0	0
Aulopiformes (lizardfish and related	Alepisauridae	*Alepisaurus ferox* ^**g**^	99	0.72	0	1.72	0
	Paralepididae	*Magnisudis atlantica* ^**g**^	99.39	0.72	0	1.72	0
Beryciformes (squirrelfish, roughies, and related)	Diretmidae	*Diretmus argenteus* ^**g,m**^	98.48–99.85	17.95	15.38	18.97	21.43
Clupeiformes (anchovies,herring and related)	Opisthoproctidae^g^	Unidentified^**g,m**^	89.72	0.72	1.92	0	0
Gadiformes (cod, grenadiers, hake and related)	Macrouridae	*Malacocephalus laevis* ^**g**^	99.84–100	0.72	0	1.72	0
	Melanonidae	*Melanonus zugmayeri* ^**g**^	99.84–99.85	0.72	0	1.72	0
Myctophiformes (laternfish)	Myctophidae	*Bolinichthys sp* ^**g**^	99.85	6.47	3.84	10.34	3.57
		*Bolinichthys indicus* ^**g**^	99.69	0.72	0	0	3.57
		*Ceratoscopelus sp* ^**g,m**^	99.69	5.76	5.77	5.17	7.14
		*Diaphus brachycephalus* ^**g**^	99.23	0.72	0	1.72	0
		*Diaphus jenseni* ^**g**^	100	0.72	0	0	3.57
		*Diaphus* sp1^**g**^	97.24	0.72	1.92	0	0
		*Diaphus* sp2^**g**^	99.69	0.72	1.92	0	0
		*Diaphus metopoclampus* ^**g,m**^	99.23	6.47	7.69	6.9	3.57
		*Diaphus rafinesquii* ^**g,m**^	99.62	6.47	1.92	10.34	7.14
		*Hygophum reinhardtii* ^**g**^	99.85	1.44	0	1.72	3.57
		*Hygophum taaningi* ^**g**^	100	1.44	3.84	0	0
		*Hygophum hygomii* ^**g**^	100	0.72	0	1.72	0
		*Lampadena chavesi* ^**g**^	99.38	1.44	1.92	0	3.57
		*Lampanyctus* ^***g***^	99.4	0.72	0	1.72	0
		*Lepidophanes* ^**g**^	100	2.16	1.92	0	7.14
		*Lobianchia gemellarii* ^***g***^	99.54–99.85	7.19	3.84	10.34	7.14
		*Notoscopelus resplendens* ^**g**^	97.21	1.44	0	3.45	0
		*Taaningichthys minimus* ^**g**^	100	0.72	0	1.72	0
		Unidentified^**g,m**^	98.25	12.23	15.38	10.34	10.71
Notacanthiformes (spiny eels)	Halosauridae	*Aldrovandia affinis* ^**g**^	99.06	0.72	1.92	0	0
Perciformes (perch and related)	Scombridae	*Naucrates ductor* ^**g,m**^	98.38	1.44	0	1.72	3.57
Stephanobercyformes	Melamphaidae	*Melamphaes typhlops* ^**g**^	99.3	0.72	0	1.72	0
		*Melamphaes sp* ^**g**^	98.77	0.72	1.92	0	0
		*Poromitra* ^**g**^	98.46	0.72	0	0	3.57
		Unidentified^**g**^	90.03	1.44	3.84	0	0
Stomiiformes (dragonfish, hatchetfish)	Gonostomatidae	*Bonapartia* ^**g**^	99.69	0.72	0	1.72	0
		*Cyclothone* ^**g**^	99.69	2.16	1.92	3.44	0
		*Gonostoma denudatum* ^**g,m**^	99.55	3.6	3.85	5.17	0
		*Margrethia obtusirostra* ^**g**^	99.5	0.72	0	1.72	0
		Unidentified^**g**^	93.19	2.16	0	3.45	3.57
	Phosichthyidae	*Vinciguerria* ^**g**^	99.69–99.85	2.16	0	3.45	3.57
	Sternoptychidae	*Argyripnus atlanticus* ^**g**^	99.85	0.72	0	1.72	0
		*Argyropelecus* ^**g,m**^	99.53–99.69	14.39	13.46	12.07	21.43
		*Sternoptyx* ^**g,m**^	98.56–99.83	27.33	32.69	17.24	39.29
		*Valenciennellus tripunctulatus* ^**g,m**^	100	0.72	1.92	0	0
	Stomiidae	*Chauliodus* ^**g**^	98.57	2.16	0	1.72	7.14
		*Stomias boa* ^**g,m**^	100	2.88	1.92	5.17	0
		Unidentified^**m**^		0.72	0	1.72	0
Syngnathiformes	Centriscidae	*Macroramphosus scolopax* ^**g,m**^		0.72	1.92	0	0
Tetradontiformes (pufferfish, sunfish and related)	Molidae	*Ranzania laevis* ^**g**^	99.84	0.72	1.92	0	0

**Figure 1:**
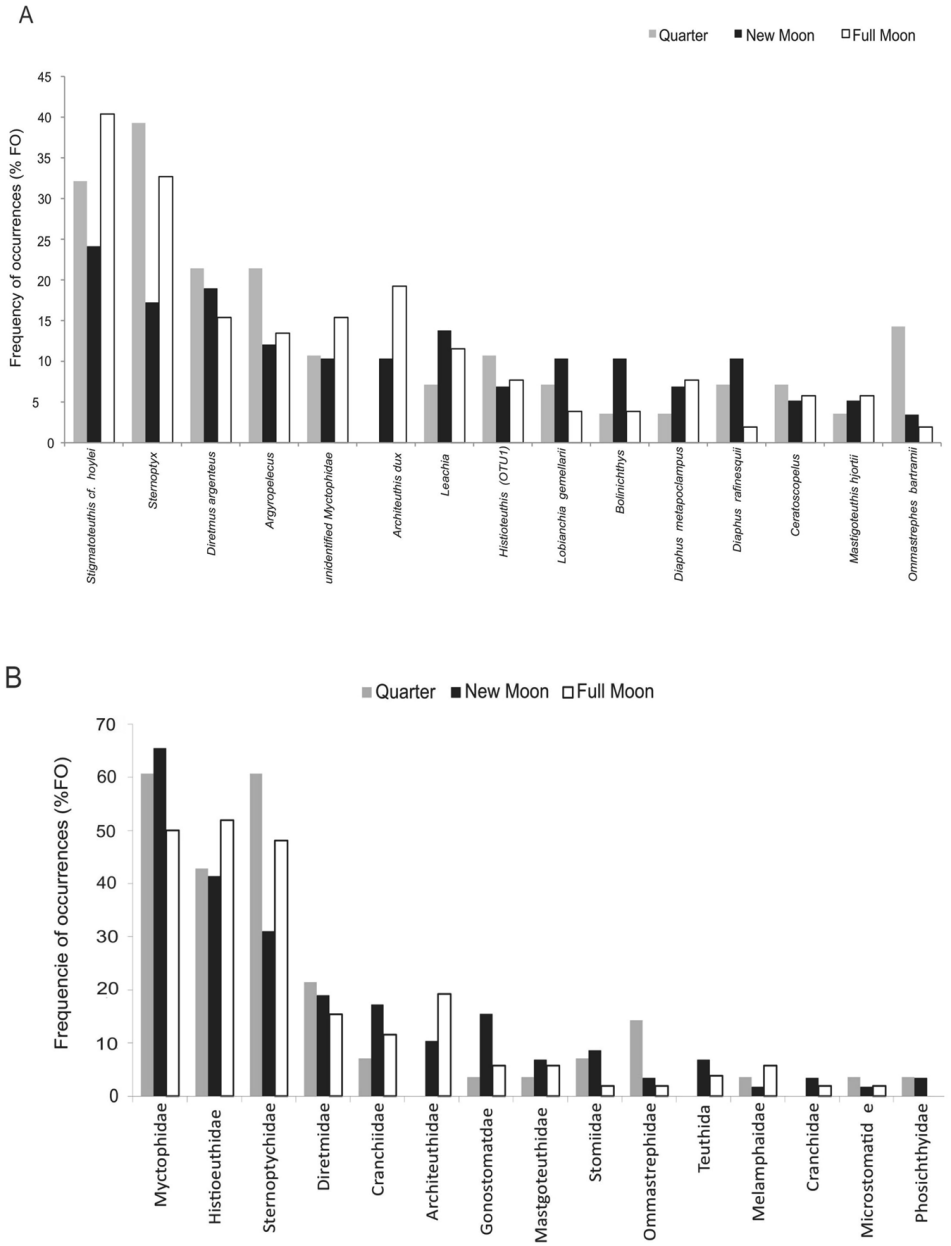
Frequency of occurrence of the taxa identified in Bulwer’s petrels stomach-contents at different lunar phases (full moon = 52, new moon = 58, quarter moon = 28); expressed as presence of a specific prey type against total number of stomach-contents collected in each lunar phase. (**A**) Prey identified to the lowest taxonomical rank. (**B**) Prey pooled into family ranks. Only the taxa occurring in over 5% of the total number of stomach contents are shown.

Taxa were identified to the lowest taxonomical rank using phylogenetic assignments of 16S rRNA barcodes (Fig. 1) and identification algorithms in BOLD-IDS for COI. Sequence similarity percentages using BOLD-IDS and BLAST are shown. The common names of representative taxa of each order are presented for teleosts. %FO, is expressed as the number of occurrences of a specific taxa divided by the total number of all stomach contents (Total) and by the total number of stomach contents collected in each lunar phase (Full Moon, New Moon, Quarter). Specimens that matched the same genera but formed distinct clusters on the tree were identified as distinct Operational Taxonomic Units (OTUs). ^g,m^Represent, respectively, genetic and morphological based methods used to make a positive identification *Query sequences were identified as *Architeuthis dux* despite low bootstrap support due to the uniqueness of this taxon and completeness of the reference tree, with sequences clustering with no other family.

### Prey composition across the lunar cycle

The main prey targeted by Bulwer’s petrels were mesopelagic teleost fish and cephalopods, dominated by myctophids, sternoptychids and histioteuthids (Fig. 1). Myctophids represented the highest diversity of prey, with 20 different prey types identified, and were also the main prey group consumed. Histioteuthids and sternoptychids, were mainly represented by two taxa, *Stigmatoteuthis* cf. *hoylei* and *Sternoptyx sp*., which were by far the most frequently eaten species (Fig. 1A). We found no obvious dietary shifts related to the lunar cycle, with no clear pattern of greater numbers of species or families consumed during new moons or full moons (Fig. 1A,B). Except for two rare prey species (with Frequency of occurrences (FO) <2%), slender sunfish *Ranzania laevis* and pilot-fish *Naucrates ductor* that are probably epipelagic, all other prey are known mesopelagic species residing in deeper oceanic layers. It should be noted that the neon-flying squid, *Ommastrephes bartramii* and the long snouted lancetfish *Alepisaurus ferox*, despite residing in deep oceanic layers, are also found at the surface during the day^40, 41^.

### Influence of lunar phase on prey collected from chicks

**Figure 2:**
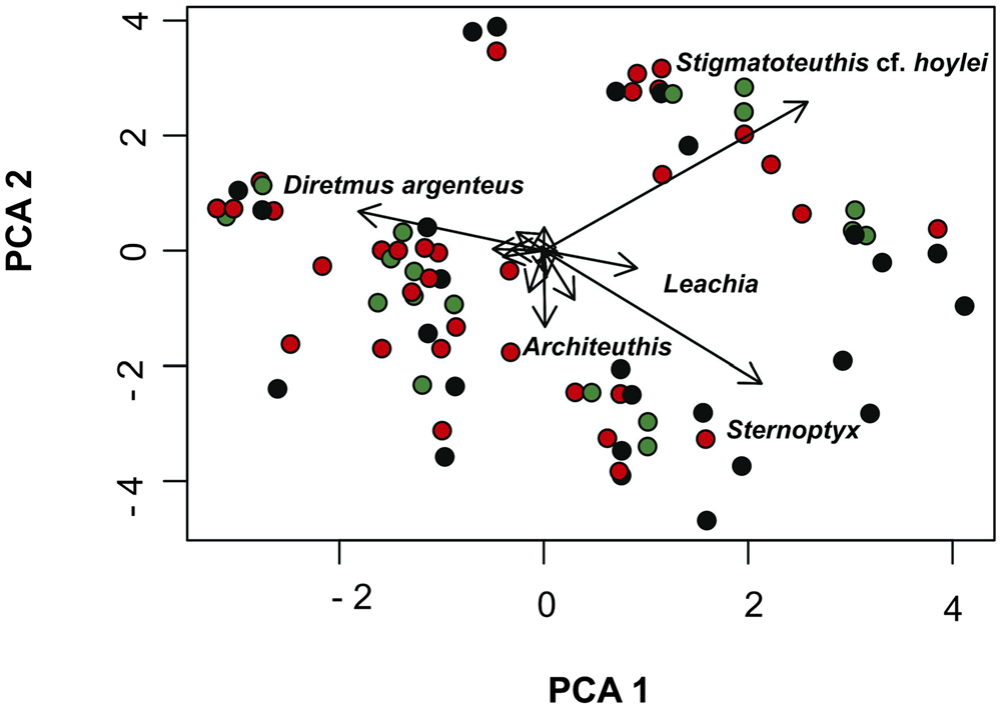
PCA scaling plot of 126 samples (after excluding rare occurrences <5%). Distances among sample points correspond to differences in species composition. Samples colored green, red and black were collected during full-, new- and quarter- moon, respectively. Total variance of PC1 = 28.12 and PC2 = 23.38 are shown on the respective axes. The magnitude of species vectors are shown on the PCA and are proportional to the variation represented by the principal components (PC1 and PC2). Only the most influential species (with eigenvalues >0.25) contributing to the co-variance of the PCA are labeled on the graph.

Principal component analysis (PCA) showed no visible distinction among samples collected at different moon phases (Fig. 2), suggesting that the moon cycle does not influence prey consumption in Bulwer’s petrel. Variation among samples was essentially marked by the presence of four prey types: hatchetfish (*Sternoptyx* sp.), cock-eyed squid (*Stigmatoteuthis* cf. *hoylei)*, spinyfin (*Diretmus argenteus*) and giant squid (*Architeuthis* sp), which were also the most dominant species found in our study (Table 1 and Fig. 2B).

We found no significant differences in the overall diet composition in relation to moon phase (perMANOVA, F_2,116_ = 0.977, R^2^ = 0.016, p = 0.479). The Shannon (H′) index of diversity of prey species did not differ significantly across moon phases (F_2,118_ = 0.012, p = 0.988). No difference in species composition correlated to the lunar phase was further detected when including the effect of other co-variables such as sampling year, site and the interaction of the lunar phase with cloud cover (perMANOVA, F_2,114_ = 1.4035, R^2^ = 0.02232, p = 0.153).

The same conclusion was obtained using only the samples collected at Deserta Grande Island during 2013, where samples collected at different moon phases showed no visible distinction on the PCA (Supplementary Fig. S3), while prey composition was not significantly correlated with moon phase (perMANOVA, F_2,70_ = 0.93033, R^2^ = 0.026, p = 0.531), or with the interaction of cloud cover with moon phase (perMANOVA, F_2,60_ = 0.72836, R^2^ = 0.02239, p = 0.680). Furthermore, moon phase had also no effect on diversity (One-way ANOVA: F_2,70_, p = 0.442), showing that these co-variables (year, island and cloud cover) were unlikely to have confounded our results.

## Discussion

Mesopelagic fauna plays a major role in marine ecosystems providing an important trophic link between surface and deep-sea communities during diel vertical migration^42, 43^. This study is the first to investigate the influence of the moon on the diet of a pelagic predator of mesopelagic prey. Our results clearly show that, contrary to expectations, differences in the levels of ambient light due to the moon cycle do not influence diet composition of Bulwer’s Petrels. This raises intriguing questions regarding the effect of lunar phases on the distribution and abundance of mesopelagic fish and squid in surface waters and how shallow-diving birds manage to capture mesopelagic prey.

Prey identification through molecular and morphological-based techniques revealed that Bulwer’s petrels feed almost exclusively on a wide range of mesopelagic species, which are known to be part of the deep scattering layers of the ocean. Previous diet studies based only on the prey item morphology, conducted on Selvagem Grande and other islands in the Northeast Atlantic, showed a similar specialization pattern where mesopelagic fish and squid were the most abundant prey of Bulwer’s petrel^36, 38^.

While the occurrence of mesopelagic species in the diet of seabirds and other surface predators has been generally related to nocturnal foraging, it is still not fully understood whether the presence of mesopelagic prey results from active predation or from scavenging of floating remains at the surface^44^. The mesopelagic prey consumed by Bulwer’s petrels are very likely captured during their vertical migrations at night, since these petrels are known to significantly increase their flight activity during darkness, with a peak after sunset when most organisms of the deep scattering layers start ascending closer to the surface^34, 35^. Moreover, the analysis of stomach contents of seabirds shot at different times of the day revealed, based on the degree of prey digestion, that Bulwer’s petrels and other avian predators of mesopelagic fish ingest their prey mostly at night^37^.

Avian predators of mesopelagic prey might cope with the putative decline of food availability near the surface during moonlit nights by switching to prey with greater epipelagic affinities (which could potentially be captured either during the day or during full moon). However Bulwer’s petrels consumed virtually no species typical of the epipelagic domain. The two very rare epipelagic prey species found remained rare throughout the lunar cycle, in one case increasing its occurrence in the diet during full moon (the slender sunfish Ranzania laevis), but in the other case showing the opposite pattern (the pilot fish Naucrates ductor). Furthermore, prey species that are thought to occur in both the mesopelagic and epipelagic realms during the day, such as the neon-flying squid, *Ommastrephes bartramii*, and the long-snouted lancetfish, *Alepisaurus ferox* ^40, 41^, were also scarce in the diet and more frequent during the new moon. Interestingly, the neon-flying squid occurs frequently in the diet of other more diurnal Procellariiformes on Selvagem Grande^31^. In the absence of a shift towards epipelagic targets during full moon, avian predators could concentrate their feeding on a few mesopelagic prey that would still come to the surface despite increased light levels. This hypothesis stems from the observations that not all mesopelagic prey behave exactly the same way^45^. For example, the bristlemouths *Maurolicus muelleri*, and *Vinciguerria nimbaria*, which are important prey of surface predatory fish, show an atypical vertical migration behaviour with schooling masses at the surface during bright nights and during the day^46, 47^. Contrary to this prediction, the diversity of prey consumed remained high across all moon phases suggesting that most mesopelagic prey species remained equally available to Bulwer’s petrel regardless of moon phase. Although it is possible that a small proportion of dietary items (regardless of moon phase) were obtained not through active predation but rather through scavenging, it is remarkable that virtually the entire community of prey detected on dark nights was also present in surface waters during moonlit nights, with over 40 taxa recorded in this study, most of which are generally reported as not occurring near the surface^48, 49^. Some particularly notorious examples include the bathypelagic species *Aldrovandia affinis* with a range at 730–2560 m^49^ and *Malacocephalus laevis*, which is not even believed to frequent the pelagic domain, being classified as bathydemersal and occurring below 200 m^49^.

It could be argued that there is perhaps a trade-off between reduced prey availability and increased visual detectability by the birds during full moons, leaving diet composition unchanged. However, visual detection of prey by Bulwer’s petrels may be actually higher during dark nights, because, with the exception of some bathypelagic fish and the squid *Architeuthis*, their prey species have multiple photophores, emitting points or patches of light at sea. Previous studies of foraging behaviour of other oceanic predators (e.g. elephant seals) have found a positive correlation between bioluminescence and foraging intensity, suggesting that these predators relied on bioluminescence of their prey to detect their occurrence^50^. It is possible that seabirds also use bioluminescence to help locate prey, but this is currently not known.

Given that most of the prey found in this study are believed to be out of Bulwer’s petrels reach during moonlit nights (over 200 m deep), we must conclude that our current understanding of their behavior, and of the vertical distribution of oceanic deep-water fish and squid, is poor. Bulwer’s petrels are shallow divers, with a maximum-recorded diving depth of 5 m^33^ which is in accordance with studies of a range of small petrels without specific diving morphological adaptations, which generally forage very close to the sea surface^51^. Although mid-water prey can be made theoretically available at the upper layers through the action of other foraging animals, such as tuna and dolphins, which herd prey to the surface, these predators (unlike Bulwer’s petrel) generally forage diurnally and show a high consumption of epipelagic prey^52^.

The responses of organisms to moonlight are, therefore, more complex than is generally thought. Even if the moon cycle plays an important role in shaping organismal distributions near the sea-surface, mesopelagic species might vary in their responses, or may be moved around by oceanographic currents (e.g. eddies)^53^ to such an extent that they are, in places, constantly locally available to pelagic predators throughout the moon cycle.

Whether specialization of surface predators on mesopelagic prey results from such physical-behavioral processes, or simply from an important fraction of mesopelagic biomass remaining irresponsive to moonlight, still needs to be properly assessed. In any case, we believe that mesopelagic organisms are far more available to surface predators than previously thought with important implications for predator-prey interactions and dynamics in many other oceanic systems.

## Material and Methods

Fieldwork was carried out at Deserta Grande (32°30′N 16°30′W), ca. 20 km SE of Madeira Island and on Selvagem Grande (30°09′N, 15°52′W), Portugal, in the Northeast Atlantic. These islands are situated approximately 270 km apart in similar deep ocean environments. To test how the lunar cycle affects prey choice of Bulwer’s petrel we collected data on the diet of the chicks.

### Ethical statement

Fieldwork was conducted so as to reduce to a minimum animal manipulation, by sampling stomach contents only once from each bird, with previous studies finding no significant effect on chick survival or growth^54, 55^. Sampling guidelines were approved by the Instituto da Conservação da Natureza e da Biodiversidade (ICNB) and by the Serviço do Parque Natural da Madeira (Portugal), carried out under the permits 2/2012S, 5/2012D and 9/2013D at Selvagem Grande and Deserta Grande. Sampling further followed the requirements of the Directive 2010/63/EU of the European Parliament and of the council for the protection of animals used for scientific purposes.

### Prey delivered to chicks

To evaluate prey composition across the lunar cycle, a total of 138 stomach contents were collected from chicks of Bulwer’s petrels at Deserta Grande during the years of 2012 (n = 25) and 2013 (n = 83) and at Selvagem Grande during 2012 (n = 30) (see Supplementary Table S1 for information on the number of samples collected in each lunar phase). A single flush of the stomach content was performed on each chick using the technique described by Wilson^56^. Samples were washed with clean water and contents filtered through a sieve to remove excess salt and preserved in 100% ethanol for molecular analysis of prey.

### DNA isolation and amplification

DNA extractions were performed on prey tissue remains collected from the stomach contents of each chick using the DNeasy Blood & Tissue kit (Qiagen)^31^. Inner tissue layers were preferentially chosen for DNA extraction, as outer tissue remains might be contaminated with DNA from other prey. Two different primer sets were used depending on the taxonomic groups targeted. For teleost fish, we amplified the widely-used cytochrome c oxidase subunit I barcode (COI)^57^ using the M13 tail primer cocktail COI-2 and PCR conditions developed by Ivanova *et al*.^58^. For cephalopods we amplified a fragment of the 16S rRNA gene using the primer set 16ar and 16br^59^ and used optimized PCR conditions^31^. The 16S rRNA gene was targeted for cephalopod DNA because the primers generated better amplification success and the sequences for this gene region had substantial better representation of different cephalopod taxa on GenBank than found for the COI barcoding region (GenBank accessed on 11-Nov-2015). PCRs were conducted with the Qiagen Multiplex PCR kit in total volumes of 12 μl and final concentrations of: 1X Multiplex PCR Master Mix, 0.25 μM of each primer and 50–100 ng/μl of DNA. PCR products were purified with the enzymes Exo I and AP (New England, Biolabs). Amplicons were sent for Sanger sequencing at Macrogen, Inc (Amsterdam, Netherlands). Chromatograms were checked for quality with BioEdit^60^. COI sequences were queried using the BOLD identification system (BOLD-IDS)^32^. Because inter-specific thresholds for 16SrRNA have not been yet comprehensively tested, especially for cephalopods, we used phylogenetic inferences for taxonomical assignment of these prey.

### Taxonomic assignments of prey

Identification of prey remains was conducted using combined morphological analyses of hard parts with molecular analyses of soft tissue. Morphological identification was only obtained for those items that had soft tissue attached to ensure, as far as possible, that only prey recently taken by Bulwer’s petrels were included in our analyses.

If no positive match could be obtained for the DNA barcode, either because there was no available reference or bad DNA quality, we identified the correspondent hard structure using morphological analysis^31^. It is important to note that for cephalopods, we obtained substantially higher numbers of tissue remains (tentacles, mantle) than fresh beaks (beaks with tissue attached). Most of the beaks obtained were very small, so that morphological identification could potentially result in incorrect species assignments^31^. Given that the number of fresh beaks was always inferior to the number of identified cephalopod species using DNA barcoding, prey estimates retrieved in this group resulted essentially from DNA barcoding.

All identifications were obtained to the lowest identifiable taxonomic rank when an exact species match could not be obtained. Molecular taxonomic identification of COI queries was based on the BOLD identification system (BOLD-IDS). Confidence in taxonomical assignments was obtained by comparing COI queries with the specimens retrieved from the BOLD database. Species ranks were assigned for those queries showing 100% of identity with public reference specimens and for a threshold of less than 2% of divergence with public references, providing these clustered monophyletically on the BOLD-IDS distance tree. Lower taxonomical ranks were assigned for queries that did not meet the above criteria, with genera and families identified following BOLD-IDS assignments.

16S mtDNA sequences (of cephalopods) were assigned phylogenetically. To construct the tree, we downloaded all available reference sequences in GenBank of all families producing positive matches in BLAST^61^ (>98% of similarity). The tree was constructed so as to include a complete list of all species occurring in the North East Atlantic for these families (see North Atlantic Register of Marine Species – NARMS at http://www.vliz.be/vmdcdata/narms/). 16S queries were condensed to haplotypes using ALTER^62^. When a reference species was missing in the GenBank database, we assigned sequences to the lowest common ancestor on the ML (maximum likelihood) tree, with the taxonomic rank assigned to a consensus lowest rank below the terminal branch of the tree. Multiple sequence alignment of queries and references were conducted in SaTé-II^63^ under MAFFT^64^, MUSCLE^65^ and FASTTREE^66^ using the GTR + γ nucleotide substitution model. Maximum likelihood tree inferences were performed in RAxML^67^ using the CIPRES Science Gateway v.3.1^68^.

### Multivariate analysis of prey composition

To assess whether lunar phase influenced the prey consumed by Bulwer’s petrel we used multivariate ordination and statistical analysis on a prey composition matrix of presence and absence data. Given that rare prey might introduce bias in multivariate analyses^69^ and mask important patterns of species composition in multivariate space, we excluded prey that occurred in less than 5% of the total number of samples from our analyses. Only samples taken within ±2 days of the corresponding lunar phase date were considered for analyses and pooled within each lunar phase category. Samples were categorized into three levels: new moon (n = 47), quarter moon (n = 27) and full moon (n = 47).

The variation in ambient light levels due to the presence and phases of the moon is of much greater magnitude than that caused by the presence of clouds^70^. However, to account for any potential effect of the presence of clouds on nocturnal light levels, we calculated a proxy for cloud cover within the vicinity of our sites during our study periods, following the procedure proposed by Wilson & Jetz^71^. In summary, cloud cover was estimated using data from the MODIS MOD09 product (Moderate Resolution Imaging Spectroradiometer, atmospherically corrected surface reflectance product), which delivers ca. 1-km ground resolution. MODIS Terra products (MOD09GA) contain several Scientific Data Sets, including a “cloud flag”, which can be extracted from bit 10 (named “internal cloud algorithm flag”) and indicates whether a given 1km-pixel is covered or not covered by clouds. We obtained daily products covering the entire study periods in 2012 and 2013 and calculated the proportion of pixels with positive values for cloud flag, within a 200 km buffer from both study colonies (see Fig. S2). This distance represents a crude estimate of the average foraging range of Bulwer’s petrels, during the chick-rearing period^72^.

Principal Component Analysis (PCA) was conducted on prey occurrence data to check for relationships between species and samples collected at different moon phases in multivariate space. PCA reduces the complexity of the original dataset by retrieving axes of maximum variance in the data, such that variation among samples can be assessed in a much smaller set of uncorrelated axes, the principal components.

Differences among lunar phases in the diet were tested using permutational multivariate analysis of variance (perMANOVA) with lunar phase as a main factor using the adonis function in R 3.1.2^73, 74^. Given that samples were collected during different years and sites we controlled the effect of these co-variables when performing perMANOVA by including them into our statistical model. Furthermore, we introduced an interaction term between lunar phase and daily cloud cover, to assess whether potential changes in moon light levels due to this co-variable significantly related to species composition.

To avoid, however, any confounding effect derived from differences between areas and/or years, we also performed all analyses as above, but including only the samples collected at Desertas islands during the year of 2013, as these were obtained during a complete lunar cycle (see Supplementary information).

Shannon indices of diversity were further obtained for each lunar phase as follows:

$${{\rm{H}}}^{{\rm{^{\prime} }}}=-\sum {p}_{i}{\rm{l}}{\rm{n}}({p}_{i})$$

where *p*_*i*_ is the proportion of species *i* in relation to the total number of prey of all species consumed and tested for significant differences among lunar phases using one-way ANOVA.

All multivariate analyses were carried out with the vegan package^74^. ﻿

### Data accessibility

Input files for phylogenetic assignments; R-codes and analysed data sets are available in Dryad (doi:10.5061/dryad.tp846). DNA sequences were deposited to GenBank (Cephalopds: KY793554-KY793618 and Teleosts: KY968099 - KY968226).

## Electronic supplementary material

Supplementary data - The diet of a nocturnal pelagic predator, the Bulwer’s petrel, across the lunar cycle
