# Supplementary material for: The diet of a nocturnal pelagic predator, the Bulwer’s petrel, across the lunar cycle
Source: Sci Rep. 2017 May 3;7:1384. doi: 10.1038/s41598-017-01312-3 (PMC5431196; doi:10.1038/s41598-017-01312-3)
Supplement: Supplementary file 1 — Supplementary data - The diet of a nocturnal pelagic predator, the Bulwer’s petrel, across the lunar cycle [file 41598_2017_1312_MOESM1_ESM.doc]

**Supplementary data**

The diet of a nocturnal pelagic predator, the Bulwer’s petrel, across the lunar cycle

Waap S ^1,2^, Symondson WOC ^1^, Granadeiro JP ^3^, Alonso H ^2, 4^, Serra-Gonçalves C ^5^, Dias MP ^2,6^, Catry P ^2^

Supplementary material includes:

1. Sample collection.
2. Phylogenetic tree of the 16S mitochondrial rRNA for identification of squid prey
3. GenBank accession numbers of the reference taxa used in 2.
4. Percentage of cloud cover variation in relation to moon phase during the study
5. Multivariate analysis using the dataset of 2013

**1. Sample collection**

Table S1. Sample collection during this study. The total number of adult birds weighted throughout a complete moon cycle, as well as the number of stomach-contents collected in chicks for each moon phase is shown, including information on sampling location and year.

**
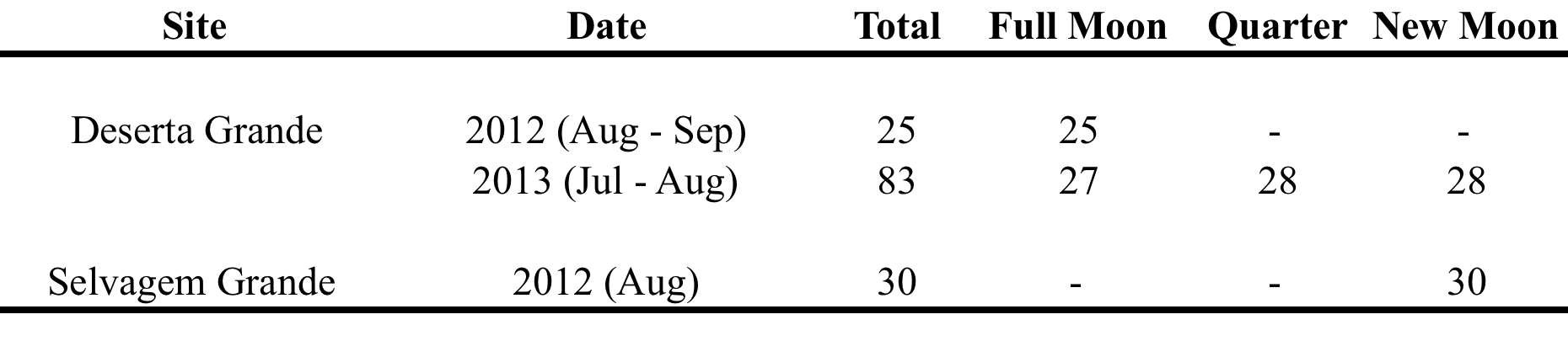
**

**2. Phylogenetic tree of the 16S mitochondrial rRNA for identification of squid prey**

**
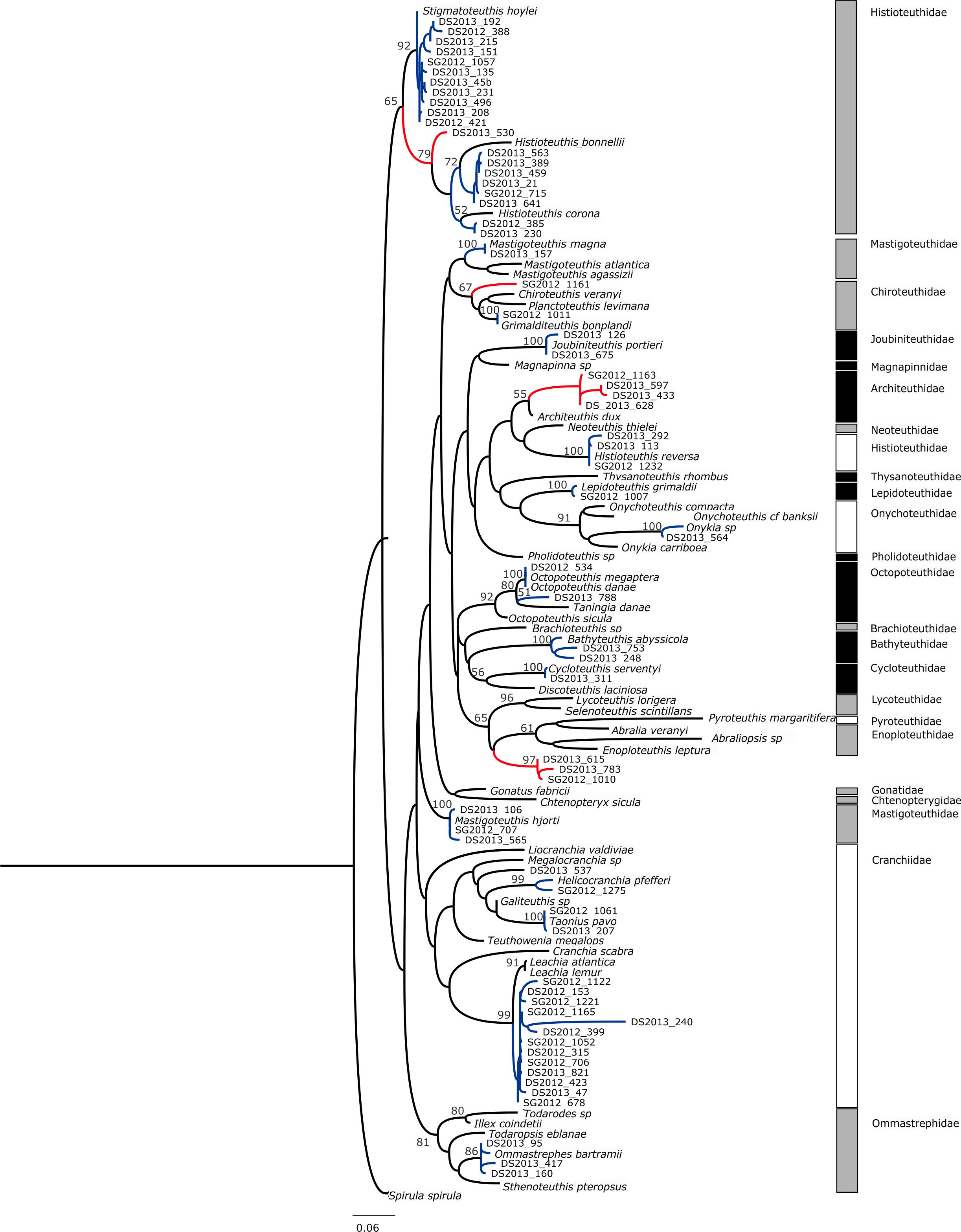
**

Fig S1. Maximum Likelihood RaxML tree to identify 16SrRNA queries of squid. Query sequences were condensed into haplotypes. The tree includes representative sequences of all families occurring in the North East Atlantic (NARMS database). Black columns show a complete taxonomic representation on the tree with all Atlantic species represented for that family; grey columns – all genera are represented on the tree; white columns – incomplete representation. Bootstrap values are shown for values > 50 on the tree. Species assignments were made for queries grouping within clusters containing all species known to occur in the North Atlantic. Queries grouping within incomplete reference clusters (for which not all North Atlantic species or genera are represented on the tree) were assigned to a consensus lowest rank below the terminal branch of the tree. If queries clustered outside a family cluster, these were not identified. Positive identifications with high support (bootstrap support values > 80) are indicated in blue and correspond to higher taxonomic rank assignments (species and genera). Red Lines indicate uncertain placement on the reference tree (low bootsrap support <80). In this case, queries were assigned only to family ranks if placed monophyletically with a reference, except for the species *Architeuthis* *dux* that was positively assigned despite a low bootstrap support = 55. Such assignment relates with its taxonomic uniqueness, as *Architeuthis dux* is the only member described for the family, with queries grouping with no other sequences. Haplotype codes are indicated on the tree showing the study site (DS, Desertas; SG, Selvagens) and the year of collection (2012, 2013). Genbank accession numbers of reference sequences are listed in Supplementary Table S2.

**3. Reference sequences accession numbers**

Table S2. Accession numbers of the reference sequences of the Maximum likelihood

| **References** | **Genbank accession numbers** | |
| --- | --- | --- |
|  |  |  |
| *Abralia veranyi* | EU735259.1 |  |
| *Abraliopsis sp* | X79595.1 |  |
| *Ancistroteuthis lichtensteinii* | EU735242.1 |  |
| *Architeuthis dux* | KC701764.1 |  |
| *Bathyteuthis abyssicola* | AJ000104.1 |  |
| *Brachioteuthis sp* | EU735223.1 |  |
| *Chiroteuthis veranyi* | EU735246.1 |  |
| *Chtenopteryx sicula* | AY293660.1 |  |
| *Cranchia scabra* | DQ280046.1 |  |
| *Cycloteuthis sirventi* | EU735204.1 |  |
| *Discoteuthis laciniosa* | EU735205.1 |  |
| *Enoploteuthis leptura* | EU735206.1 |  |
| *Galiteuthis sp* | AY616987.1 |  |
| *Gonatus fabricii* | AY681033.1 |  |
| *Grimalditeuthis bonplandi* | EU735226.1 |  |
| *Helicocranchia pfefferi* | AF110099.2 |  |
| *Histioteuthis bonellii* | EU735248.1 |  |
| *Histioteuthis corona* | EU735211.1 |  |
| *Histioteuthis reversa* | EU735256.1 |  |
| *Illex coindetii* | AY616985.1 |  |
| *Joubiniteuthis portieri* | EU735213.1 |  |
| *Leachia atlantica* | EU735203.1 |  |
| *Leachia lemur* | EU735252.1 |  |
| *Lepidoteuthis grimaldii* | DQ280048.1 |  |
| *Liocranchia valdiviae* | X79587.1 |  |
| *Lycoteuthis lorigera* | EU735257.1 |  |
| *Magnapinna sp* | EU735227.1 |  |
| *Mastigoteuthis agassizi* | EU201158.1 |  |
| *Mastigoteuthis atlantica* | KC861001.1 |  |
| *Mastigoteuthis hjorti* | KC860987.1 |  |
| *Mastigoteuthis magna* | EU201156.1 |  |
| *Megalocranchia sp.* | EU735228.1 |  |
| *Neoteuthis thielei* | EU735215.1 |  |
| *Octopoteuthis danae* | EU735266.1 |  |
| *Octopoteuthis megaptera* | EU735258.1 |  |
| *Octopoteuthis sicula* | EU735217.1 |  |
| *Ommastrephes bartramii* | HQ829182.1 |  |
| *Onychoteuthis cf. banksii* | EU735250.1 |  |
| *Onychoteuthis compacta* | AJ223482.1 |  |
| *Onykia carriboea* | EU735234.1 |  |
| *Onykia sp* | AJ223486.1 |  |
| *Pholidoteuthis adami* | EU735254.1 |  |
| *Planctoteuthis levimana* | EU735247.1 |  |
| *Pyroteuthis margaritifera* | EU735209.1 |  |
| *Selenoteuthis scintillans* | EU735230.1 |  |
| *Spirula spirula* | AJ966785.1 |  |
| *Sthenoteuthis pteropus* | AB270957.1 |  |
| *Stigmatoteuthis cf hoylei* | DQ280047.1 |  |
| *Taningia danae* | AY393901.1 |  |
| *Taonius pavo* | KC603481.1 |  |
| *Teuthowenia megalops* | AY616984.1 |  |
| *Thysanoteuthis rhombus* | AB191135.1 |  |
| *Todarodes filippovae* | AB270950.1 |  |
| *Todaropsis eblanae* | AY616988.1 |  |
|  |  |  |
|  |  |  |

**4. Variation of the percentage of cloud cover in relation to moon phase**


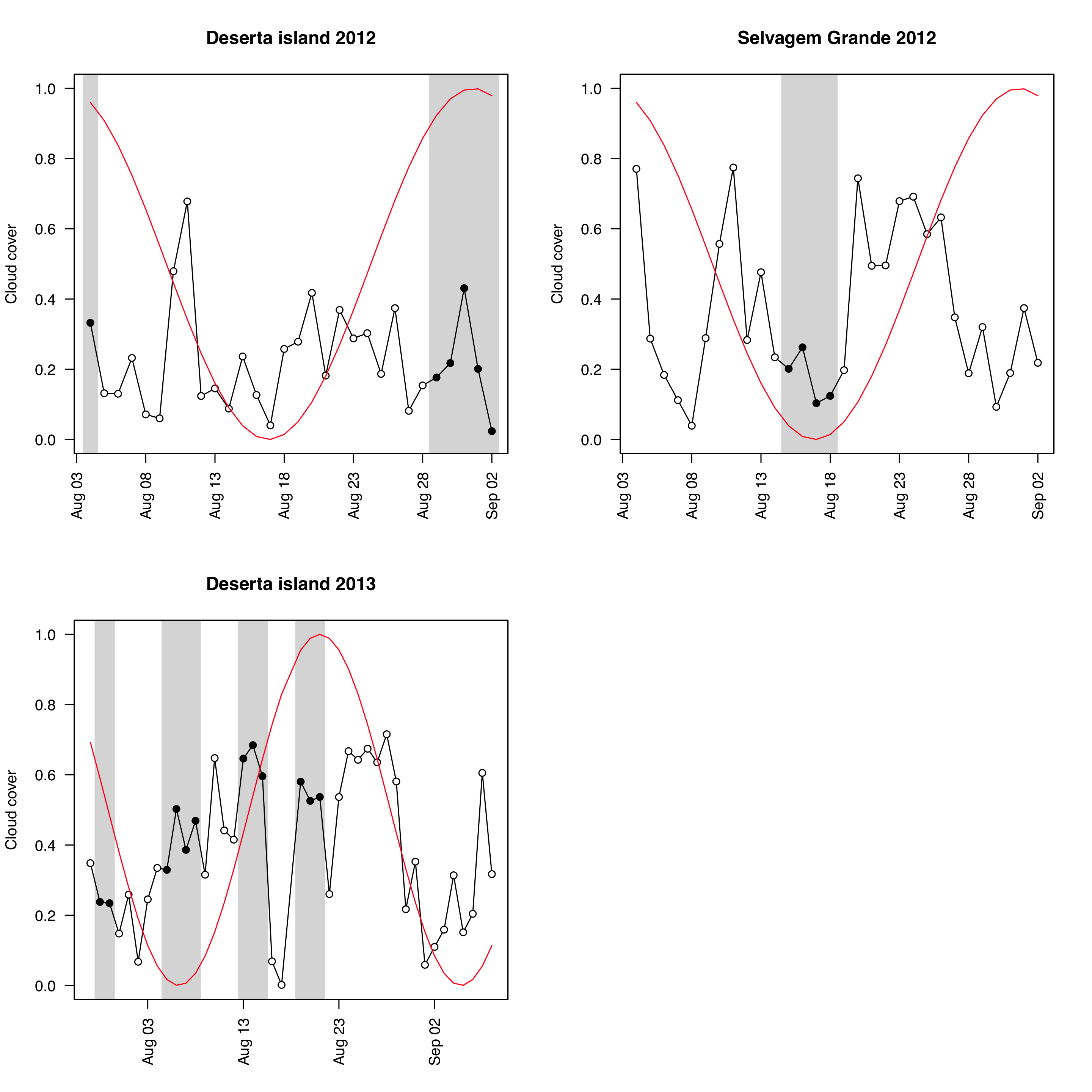


Fig S2. Lunar phase (0= new moon; 1= full moon) and cloud cover(proportion of cells with cloud flag=1 within a 200 buffers from thestudy colonies, for explanation see Methods) during the study period inDeserta and Selvagem Islands. The periods used in the analysis of the effects of moon and cloud cover on diet are shaded grey

**2. Multivariate analysis on the diet of chicks using the Deserta Grande 2013 dataset**

**Methods**

To test the effect of the moon phase on prey occurrences of Bulwer’s petrels we performed multivariate ordination and statistical analyses (PCA and perMANOVA) on 83 dietary samples collected at Deserta Grande during 2013. All multivariate analyses were performed on a matrix of prey occurrences containing samples collected during three different moon phases: new moon, quarter and full moon. Only taxa with occurrences > 5% were considered for multivariate analyses.

**Results**

PCA ordination showed no visible patterns related with the moon phase (Figure S1). Distances among samples were essentially explained by the presence of 3 different prey types: *Histioteuthis*, *Sternoptyx* and *Diretmus*, but occurrences of these were not related with any specific moon phase. perMANOVA tests showed no significant differences in prey composition among samples collected at different moon phases and its interaction with cloud cover (F2,60= 0.728, R2=0.022, p= 0.680). Results obtained using multivariate analysis agreed with Shannon index of diversity, with no significant differences in prey diversity across different moon phases (One-way ANOVA: F_2, 70_ , p= 0.442).


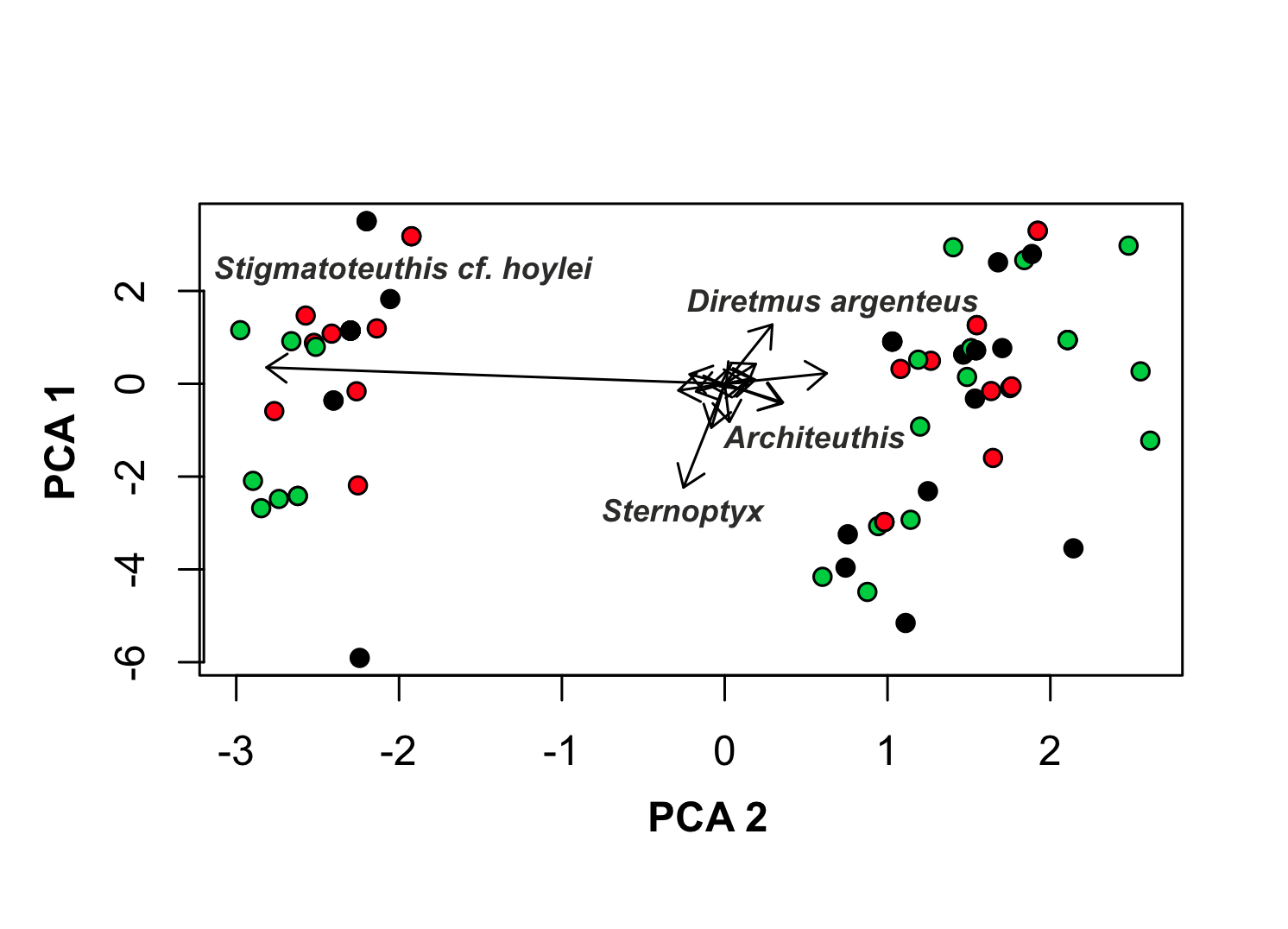


Fig. S3. PCA scaling plot of 74 samples (after excluding rare occurrences < 5%): full_moon (n=26), quarter moon (n=27), new moon (n=21). Distances among sample points correspond to differences in species composition. Samples colored green, red and black were collected during full-, new- and quarter- moon, respectively. The magnitude of species vectors are shown on the PCA and are proportional to the variation represented by the principal components (PC1 and PC2). Total variation of PC1 and PC2 is shown as percentages on the respective axes. Co-occurrences of the four most influential species (with eigenvalues > 0.25) contributing to the co-variance of the PCA are labeled on the graph.
